# Supplementary material for: ERASE: a novel surface reconditioning strategy for single-molecule experiments
Source: Nucleic Acids Res. 2018 Nov 20;47(3):e14. doi: 10.1093/nar/gky1168 (PMC6379648; doi:10.1093/nar/gky1168)
Supplement: Supplementary Data [file nar_47_3_e14_s3.pdf]

Supplementary Information

Supplementary Table 1: Oligonucleotide sequences used in this study.

Anchor 1	/BioTEG/CACTGGTGTT
Tether 1	CA/Cy3/ATTAAAATTCCGACAACACCAGGT
Switch 1	ACCTGGTGTTGTCGGAATTTTAAT
Control Switch 1	GAGTGGGGAGTCAAAGTAAATTCAAAACCAGGACTCACTGCGAGGTA
Anchor 2	TCAATTCGTCGTC/BioTEG/
Tether 2	/Cy3/GACGACGAATTGAAGTGAAA
Switch 2	TTTCACTTCAATTCGTCGTC
RNA Tether 2	/Cy5/GACGACGAAUUGAAGUGAAA
DNA Anchor-70S	AGTTTTAGGTTGCCCCCCTTTTTTTTTTTTTTTTTTTTTTTTTTTTT<br>T/BioTEG/
mRNA-70S	CAACCUAAAACUUACACACCCUUAGAGGGACAAUCGAUGUUCAAAGU<br>CUUCAAAGUCAUC
Switch-70S	GGGTGTGTAAGTTTTAGGTTG
Orthogonal Tether	/Cy5/GACGACGAATTGATCACTTT
Orthogonal Switch	AAAGTGATCAATTCGTCGTC

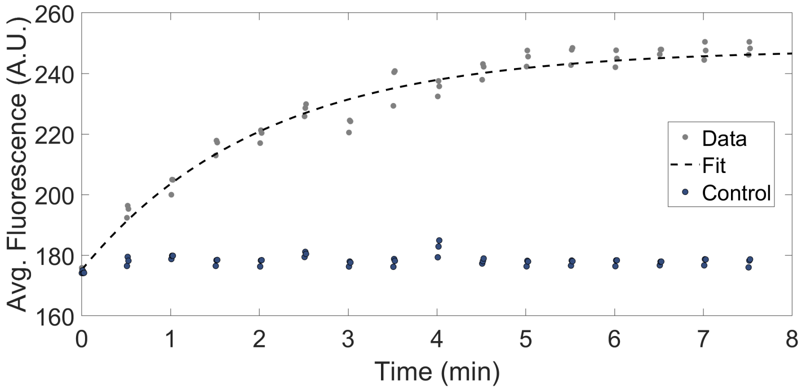

Supplementary Figure 1: Mean fluorescence from Cy3 labeled DNA binding to a PEGylated surface. To avoid photobleaching the laser is strobed for 2 second intervals every 30 seconds at an exposure time of 500 ms. The data is fitted with a single exponential to obtain a rate of anchor hybridization. As a control, Cy3 labeled DNA is introduced to a surface without anchor molecules.

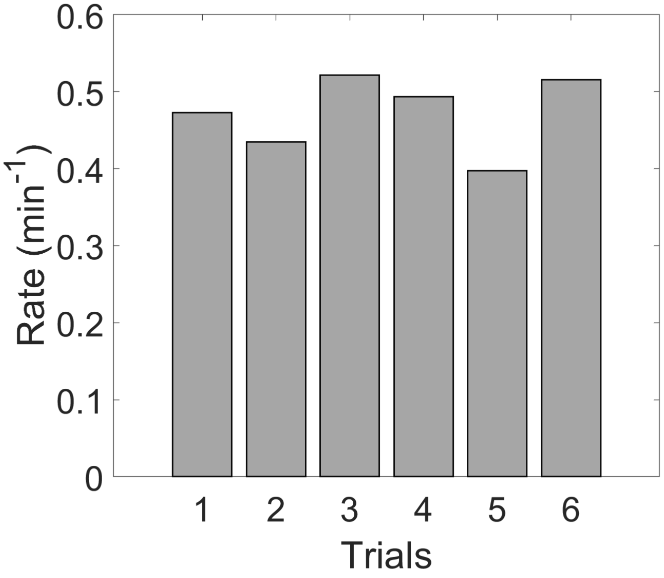

Supplementary Figure 2: Rate of mean fluorescence increase over rounds of ERASE. Similar rate constants were extracted from single exponential fits of anchor hybridization for several consecutive rounds of ERASE.

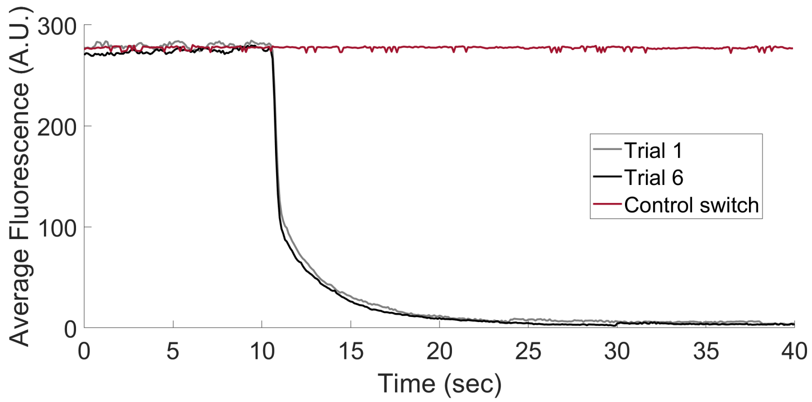

Supplementary Figure 3: Time-dependent fluorescence for Cy3 labeled DNA after introducing switch. The switch molecule is introduced at 10 seconds. Decrease in fluores- cence is complete in tens of seconds and is similar from Trial 1 to Trial 6. A control switch with a different sequence does not engender a change in fluorescence.

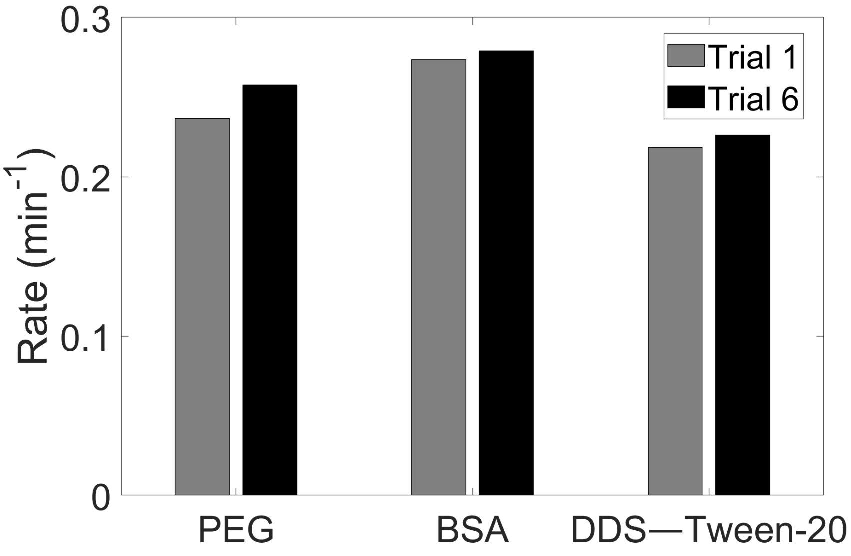

Supplementary Figure 4: Rate of mean fluorescence increase for Cy3 labeled DNA across different surface passivation schemes. Similar rate constants were extracted from single exponential fits of anchor hybridization for the first and sixth round of ERASE for three surface passivation techniques: 1) PEG, 2) BSA, 3) DDS–Tween-20+BSA.

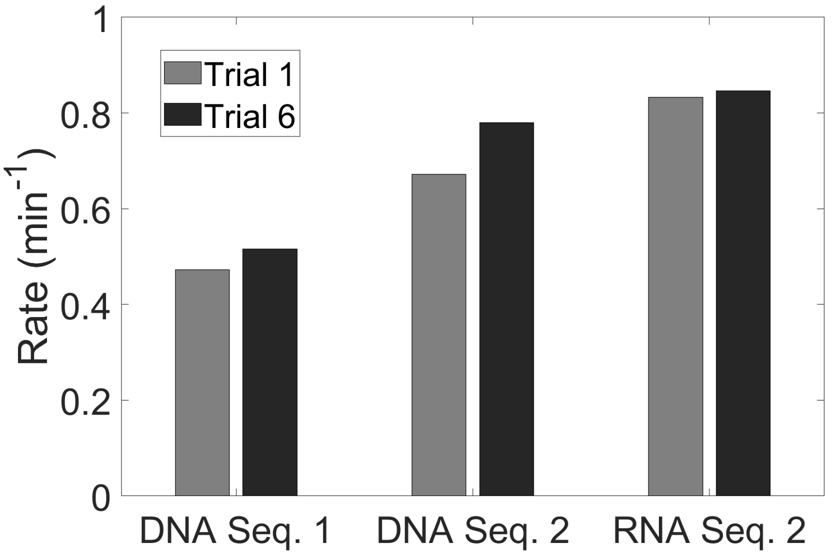

Supplementary Figure 5: Rate of mean fluorescence increase for different sequences of Cy3 labeled DNA and Cy5 labeled RNA. Similar rate constants were extracted from single exponential fits of anchor hybridization for the first and sixth round of ERASE for different nucleic acids: 1) DNA with internally labeled Cy3, 2) DNA with a different sequence and end labeled with Cy3, 3) RNA with the same sequence as 2) and end labeled with Cy5.

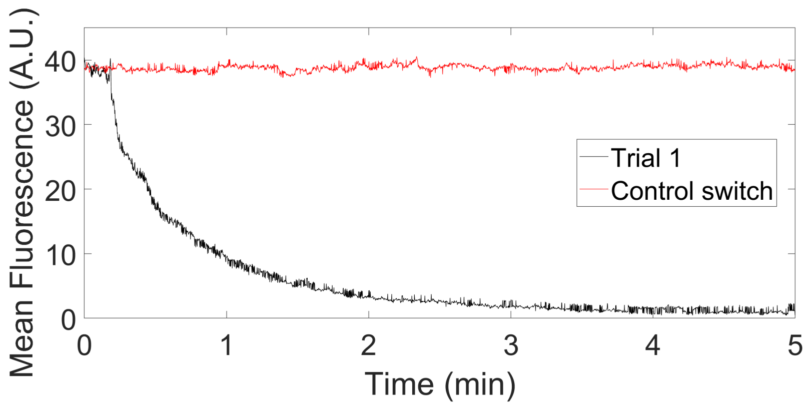

Supplementary Figure 6: Time-dependent fluorescence for 70S complexes after introducing switch. The switch molecule is introduced at 10 seconds. Decrease in fluores- cence is complete in a few minutes. A control switch with a different sequence does not cause a change in fluorescence.
